# P21 and P27 promote tumorigenesis and progression via cell cycle acceleration in seminal vesicles of TRAMP mice

**DOI:** 10.7150/ijbs.35092

**Published:** 2019-08-19

**Authors:** Tonghui Li, Fangfang Wang, Yanmei Dang, Jiajie Dong, Yu Zhang, Chi Zhang, Ping Liu, Yanhong Gao, Xiaojun Wang, Sijun Yang, Shan Lu

**Affiliations:** 1 School of Life Sciences, Jiangsu Key Laboratory for Molecular and Medical Biotechnology, Nanjing Normal University, Nanjing, 210023, China;; 2 ABSL-3 Laboratory at the Center for Animal Experiment and State Key Laboratory of Virology, Wuhan University School of Medicine, Wuhan, 430071, China.

**Keywords:** tumorigenesis, transgenic adenocarcinoma mouse prostate, epidermal growth factor receptor

## Abstract

Transgenic adenocarcinoma mouse prostate (TRAMP) model is established to mimic human prostate cancer progression, where seminal vesicle lesions often occur and has been described as phyllodes-like epithelial-stromal tumors. However, the molecular mechanism regulating tumorigenesis and progression in seminal vesicles of TRAMP mice remains largely unknown. In this study, C57BL/6 TRAMP mice were found to have a significantly shorter lifespan than wild-type (WT) mice and all of the seminal vesicles were markedly increased in size and weight with age from 24 weeks exhibiting a clearly papillary-phyllode pattern, though no obvious difference was observed in multiple organs including heart, liver, spleen, lungs, kidneys, testicles and bone between TRAMP and WT mice, and less than 10% of TRAMP mice developed prostate tumors. Western blotting showed Cyclin (CCN) B1 and CCND1 were remarkably overexpressed in seminal vesicle tumors of TRAMP mice at 24 weeks of age and increased with age till the end of trial, which was confirmed by Immunohistochemistry (IHC). P21 and P27 were also significantly augmented, whereas P53 and phosphorylated P53 (p-P53) were constantly expressed in normal controls and P53 did not appear to be mutated. Not only cyclin-dependent kinase (CDK) 1 and phosphorylated forkhead box protein (FOX) O1 but also CDK4, CDK6 and phosphorylated retinoblastoma-associated protein (RB) had similar increase trends, so did epidermal growth factor receptor (EGFR), AKT serine/threonine kinase (AKT), and their respective phosphorylation levels. Signal transducer and activator of transcription (STAT) 3, p-STAT3, enhancer of zeste homolog 2 (EZH2) and EZH2 mediated trimethylation of histone H3 lysine 27 (H3K27me3) were considerably elevated, too. Taken together, this finding suggests P21 and P27 promote carcinogenesis and development in seminal vesicles of TRAMP mice via accelerating cell cycle progression, in which oncogenic transformation of P21 and P27 might be through regulation of EGFR-AKT signaling.

## Introduction

Transgenic adenocarcinoma mouse prostate (TRAMP) model is constructed to mimic human prostate cancer progression ranging from mild intraepithelial hyperplasia to large multinodular malignant neoplasia with distant site metastasis by using the prostate epithelia-specific rat probasin promoter (-426/+28 bp) to drive expression of simian virus 40 large tumor antigen-coding region (Tag) which induces oncogenic progression after binding to and inactivating tumor suppressors P53 and retinoblastoma associated protein (RB) 1, 2. However, the consequent lesions are not merely restricted to prostate. Seminal vesicles, the androgen-dependent accessory sex glands from the male reproductive system, often become dilated, even generate large mass lesions overgrowing prostate, which has been identified as phyllodes-like epithelial-stromal tumors mainly from stromal cell proliferation 3-5. Early seminal vesicle tumors were not regarded to be conclusively malignant 5. Later, it was considered to be malignant after metastatic tumors were recognized to be similar to seminal vesicle tumors without epithelial differentiation and carcinosarcomatous patterns in TRAMP mice by morphological analysis and pathological diagnosis 6. Interestingly, the origin of seminal vesicle tumors in TRAMP mice is still controversial. Some supposed that seminal vesicle tumors in TRAMP mice resulted from local extension and invasion of prostate tumors, which could be a good model for studying seminal vesicle lesions in association to prostate 3, 4, 7, 8. The other considered that seminal vesicle tumors arose from a completely different origin compared to prostatic tumors, belonging to extra-prostatic transgene-positive lesions like primary anaplastic tumors in the midbrain, poorly differentiated adenocarcinomas of submandibular salivary gland, etc. of TRAMP model 5, 9. Moreover, the molecular mechanism regulating tumorigenesis and tumor progression in seminal vesicles of TRAMP mice remains largely unknown.

In the present study, we characterized phenotypical features of TRAMP mice, especially compared the expressions of a series of important genes to regulate cell cycle and cell proliferation including cyclin (CCN) B1, CCND1, P53, P21, P27, epidermal growth factor receptor (EGFR), AKT serine/threonine kinase (AKT), **s**ignal transducer and activator of transcription (STAT) 3 and enhancer of zeste homolog 2 (EZH2) between seminal vesicles of TRAMP mice and normal controls at different ages. We found P21 and P27, mediated by EGFR-AKT signaling, promoted cell cycle progression in tumor occurrence and development in seminal vesicles of TRAMP mice while WT P53 function was lost, which potentially expanded and deepened the understanding of the preclinical model TRAMP of human prostate cancer.

## Materials and methods

### TRAMP mice

Hemizygous male mice harboring the rat probasin--Tag (C57BL/6-Tg (TRAMP) 8247Ng/J) and wild type C57BL/6 females were obtained from the Model Animal Research Center of Nanjing University (MARCNU, Nanjing, Jiangsu, China) for breeding. Tail genomic DNA form each newborn mice at the time of weaning was isolated in lysis buffer (50 mmol/L Tris-HCl, pH8.0; 100 mmol/L EDTA, pH8.0; 0.5% SDS) with proteinase K (Merck KGaA, Darmstadt, Germany). Genotyping was carried out by PCR following MARCNU's protocol. All animal experiments were conducted in accordance with institutional guidelines and approved by the Institutional Animal Care and Use Committee of Nanjing Normal University.

### Phenotypical analysis

Transgenic male offsprings were divided into several groups from 12 weeks of age till 48 weeks with a 6-week interval (n = 8~10/group) and the nontransgenic littermates at the same age were used as controls (n = 5/group). When mice in one group grew up to desired age, they were weighted individually and anaesthetized with ether for sacrifice to isolate the main organs including heart, liver, spleen, lung, kidneys, testicles and seminal vesicles. Every kind of organs was washed with 1XPBS (-) and weighted, separately. The seminal vesicle tissues from more than 3 mice of each group were immersed in 4% paraformaldehyde at 4°C overnight, and rinsed in 50% ethanol, dehydrated with a serial ethanol gradient to be finally embedded in paraffin wax blocks, respectively. The samples were then cut into 5 μM-thick tissue sections and placed on the microscopic glass slides for performing hematoxylin and eosin (H&E) staining to check morphological change after deparaffinization and rehydration. Other tissues were immediately stored in -70 ℃ for later protein extraction. *X*-ray photograph was captured by Multispectral FX Pro In-Vivo Imager from Carestream Health (Rochester, NY, USA) as soon as mice were anaesthetized with 4% chloral hydrate at the dosage of 1 ml /100 g. In addition, the time and number of sudden death in mice was also recorded during the experimental period.

### Western blotting and Immunohistochemistry (IHC)

The homogenized mouse tissues were lysed in ice-cold RIPA buffer for Western blotting as detailed in our previous paper 10. Paraffin sections of mouse tissues submitted to IHC were prepared similarly as in phenotypical analysis. Antigen retrieval was performed by incubation in 0.01 mol/L citrate buffer (pH 6.0) and maintained at a sub-boiling temperature for 10 minutes in microwave. Blockage of endogenousperoxidases was carried out with 3% hydrogen peroxide for 10 minutes and followed by an incubation of 3% BSA in PBS for blocking at room temperature for 1 h. Primary antibodies were added to each section for overnight incubation at 4°C, and the sections were washed and appropriately incubated with biotin-labeled secondary antibodies in compliance with the protocol of ImmunoCruz mouse/rabbit ABC Staining System (Santa Cruz Biotechnology, Santa Cruz, CA, USA). The immunoreactivity of antibodies was evaluated using the brown DAB precipitate, and the counter-stainings were performed with hematoxylin. Then the slides were dehydrated, mounted in the inverted microscope DMi8 (Leica Microsystems CMS GmbH, Wetzlar, Germany). Images were captured on a Leica DFC7000T camera via Leica Application Suite software (Version 4.7.1). Antibodies against CCNB1, CCND1, AKT, p-AKT (Ser473), p53, p21 and EGFR were obtained from Santa Cruz Biotechnology. Antibodies for p27, CDK4, CDK6, EZH2, STAT3, anti-mouse peroxidase-conjugated secondary antibody were purchased from Proteintech Group (Rosemont, IL, USA). Antibodies against p-STAT3 (Tyr705), trimethylation of histone H3 lysine 27 (H3K27me3) and p-P53 (Ser15) were purchased from Affinity Biosciences (Cincinnati, OH, USA). Anti-p-EGFR (TyR1173), anti-CDK1 and anti-p-RB (Ser780) antibodies were obtained from Bioworld Technology (Louis Park, MN, USA). Antibody for p-FOXO1 (Ser249) was purchased from Absin Bioscience (Shanghai, China). The peroxidase-conjugated secondary antibody for rabbit was purchased from Cell Signaling Technology (Boston, MA, USA).

### Statistics

Data were analyzed using GraphPad Prism 5 (GraphPad Software, Inc., La Jolla, CA, USA) for comparison of body weight between TRAMP and WT mice with Student's *t* test, and for comparison of survival between TRAMP and WT mice with Log Rank (Mantel-Cox) test. Differences were considered to be statistically significant if *P*-value was <0.05 (*P <0.05, **P < 0.01, *** *P* < 0.005).

## Results

### C57BL/6 TRAMP mice have a significantly shorter lifespan compared to normal controls

**Figure 1:**
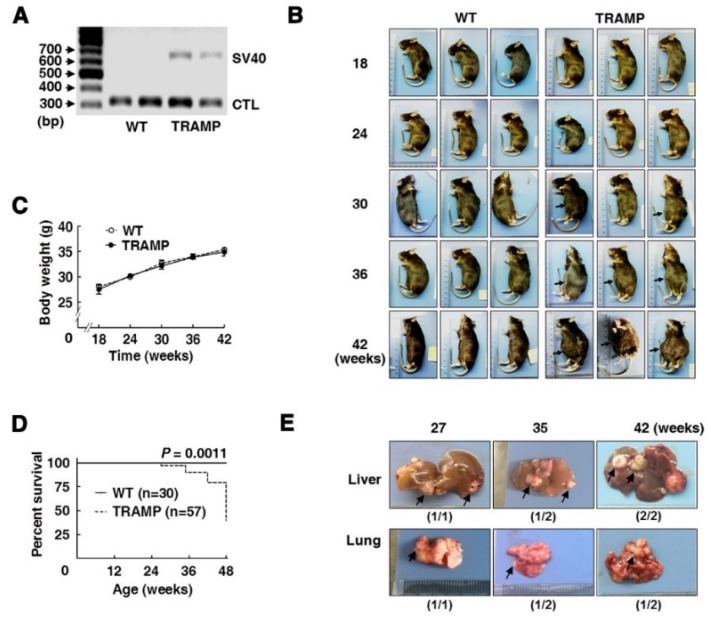
** Phenotypic features and mortality of transgenic adenocarcinoma of mouse prostate (TRAMP) mice. (A)** Gel electrophoresis of PCR products for offspring genotyping and **(B)** mouse appearances of different age-groups with gradual swelling of lower abdomen (arrow). Comparison of **(C)** body weight and **(D)** survival rate between TRAMP and WT mice of the same ages using Graphpad Prism 5. Data were shown as means ±SEM of 5 to 10 mice per group for body weight, and unpaired Student's *t* tests were applied to statistical analysis (*P* > 0.05). Survival curves were analyzed for significant differences by Log Rank (Mantel-Cox) test (***P<0.005). **(E)** Representative metastatic nodules (arrow) on liver and lungs of TRAMP mice undergoing sudden death before 48 weeks old.

Mouse offsprings at 3 weeks old were carried out for genotypic identification by PCR. After gel electrophoresis, TRAMP mice were identified by one band at 650 bp corresponding to specific amplification of Tag fragment, and the other band at 324 bp was used as an internal control (Fig. 1A). Then offspring male mice were isolated and randomly grouped according to experimental arrangements. As mice grew up, no obvious difference was observed in physical appearance and body weight between TRAMP and WT mice of the same ages, except that swollen abdomen occurred in about two thirds of TRAMP mice from 30 weeks or so, and it became universal and increasingly severe with age in TRAMP mice (Fig. 1B and C). The first sudden death appeared at 27 weeks of age in a total of 57 TRAMP mice before scheduled sacrifice and the others happened at 35, 42 and 48 weeks of age responding to different numbers of 2, 2 and 5 in TRAMP mice, separately, while no abnormal death was observed in all of WT mice. The median survival time of TRAMP mice was calculated to be 48 weeks by survival curves (Fig. 1D), suggesting the difference in mortality rate between TRAMP mice and normal mice was extremely significant (*P* = 0.0011). Nodular metastasis was found in the liver and lungs of 4 (80%) and 3 (60%) among total 5 TRAMP mice which died before 48 weeks old, respectively (Fig. 1E), indicating that lifespan shortening in TRAMP mice might result from tumor metastasis in liver and lungs. It was reported that C57BL/6 TRAMP mice developed palpable tumors in the pelvis at 10-38 weeks of age and most died from 24 to 40 weeks of age 3, 11. The results displayed phenotypical variation of C57BL/6 TRAMP mice basically consisted with previous reports except for a slower tumor progression, which was probably due to sublineage difference 5, 7.

### Multiple organs of TRAMP mice in morphology and weight are not obviously different from those of normal mice within 42 weeks of age

**Figure 2:**
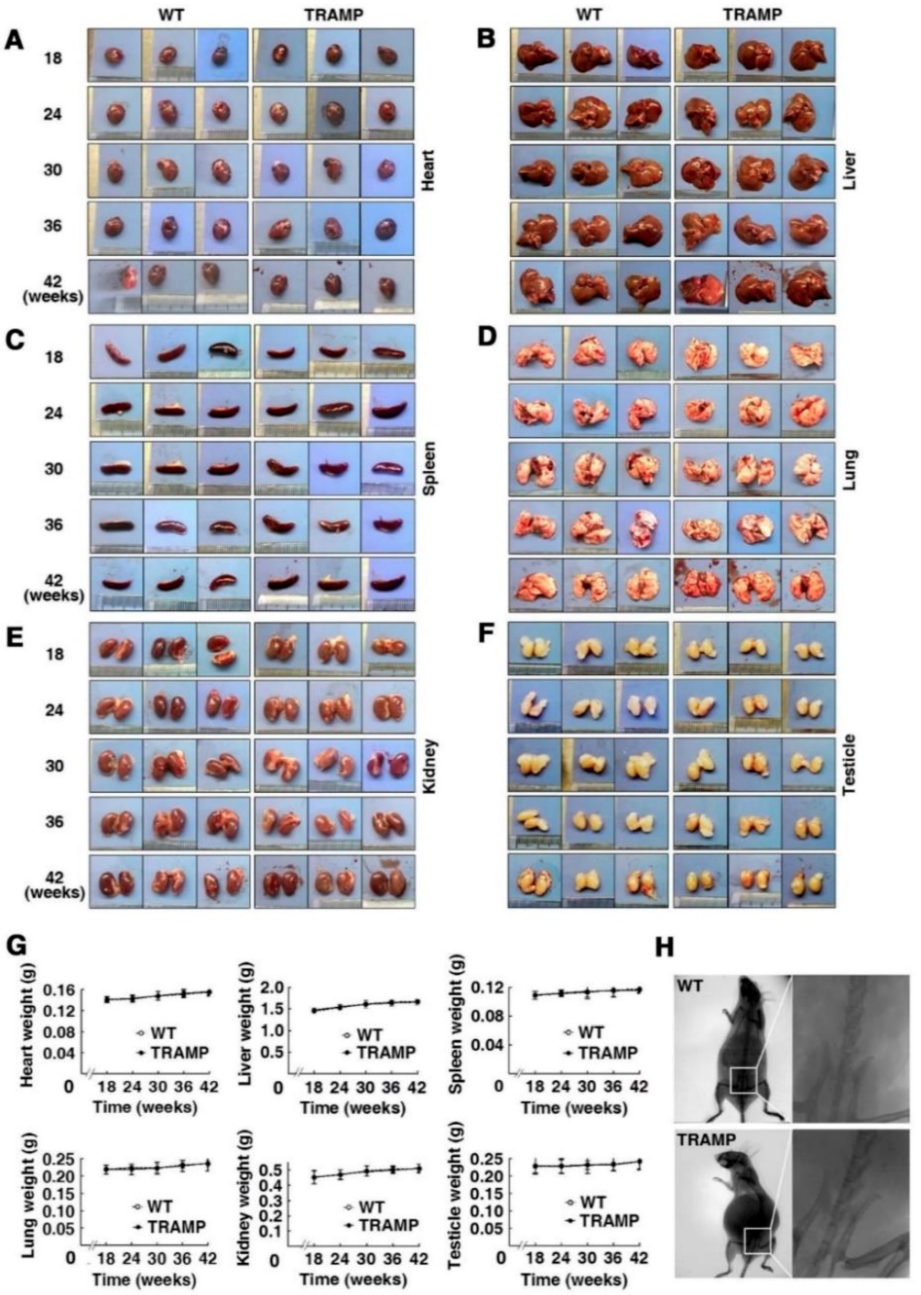
** Morphologic analysis of various internal organs of TRAMP mice at different age gro**ups. Appearances of hearts **(A)**, livers **(B)**, spleens **(C)**, lungs **(D)**, kidneys **(E)** and testicles **(F)** from 3 mice in TRAMP or WT groups were represented for each experimental group, which were weighted and shown as (G) means ±SEM of every kind of organs per mice group for statistical analysis with unpaired student's t tests (P > 0.05). (H) Bone images from representative mice were obtained by *X*-ray photography as detailed in text.

To check whether the internal organs of TRAMP mice have phenotypical change compared to WT mice, hearts, livers, spleens, lungs, kidneys and testicles of each group mice were examined based on shape, color, size and weight. No apparent difference in appearance and weight was found in every kind of these organs between TRAMP and WT mice of the same ages (Fig. 2A-F). The six kinds of organs slightly gained weight during the period of mouse growth from 18 to 42 weeks, but there is little difference in weight for each kind of these organs between TRAMP and WT mice at the same ages (Fig. 2G). *X*-ray photograph showed no remarkable change in bone morphology and structure, particularly in pelvic cavity in a 42 week-old TRAMP mouse compared to normal control (Fig. 2H). Indeed, bone lesions were also not detected in a 34-week-old C57BL/6 TRAMP mouse, whereas bone metastasis with characteristic osteolytic histology was observed in a single [TRAMP×FVB] F1 mouse at 22 weeks of age 2. This might be due to difference in genetic background since several articles have reported that tumor progression was faster in TRAMP mice with FVB background than pure C57BL/6 12, 13.

### TRAMP mice develop seminal vesicle tumors resulting from up-regulation of CCNB1 and CCND1

**Figure 3:**
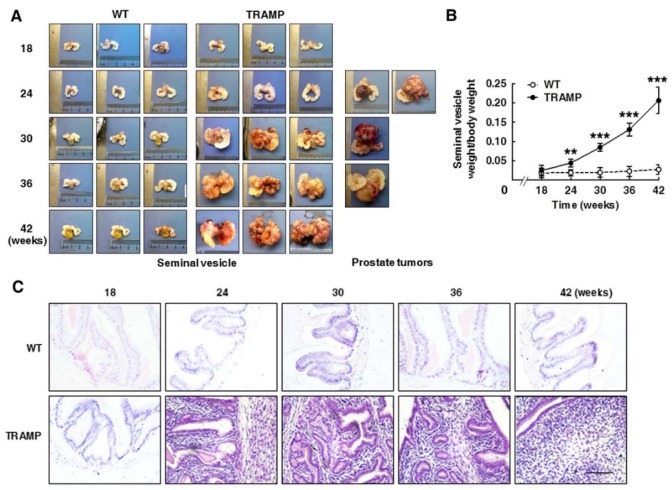
** Tumorigenesis and progression in seminal vesicles of TRAMP mice. (A, left)** Appearances of seminal vesicles from 3 mice in TRAMP or WT groups were represented for each experimental group and **(A, right)** prostate tumors were showed from all experimental TRAMP mice. **(B)** Seminal vesicles from different age groups of TRAMP mice were weighted and the data were shown as means ±SEM of at least 3 mice per time point for statistical analysis between TRAMP and WT mice with unpaired student's t tests (**P<0.01, ***P<0.005). (C) Paraffin sections of seminal vesicles were used for H&E staining to compare the differences between TRAMP and WT mice (Scale bar: 50 μm).

Seminal vesicles in TRAMP males were visibly larger than in WT mice at 24 weeks of age, and were continuously enlarged with age in the period of mouse growth. Tumor masses clearly protruded on seminal vesicle surfaces of TRAMP mice from 24 weeks of age and gradually became profound seminal vesicle obstruction. The incidence of seminal vesicle tumors was 100% at 24 weeks (Fig. 3A). Interestingly, a more spherical, highly vascularized prostatic tumor engulfing bladder and urethra was detected in only 4 cases (9.5%) among a total of 42 TRAMP mice in various age groups used for sacrifice, of which 2 cases belonged to 24 week age group, and each one case was at 30 and 36 week group, respectively. Indeed, TRAMP mice containing FVB background were more preferably to develop spherical prostate tumors 11, 13. The average weight of seminal vesicles in TRAMP mice became more than double from 18 to 24 weeks of age and were increased by over 8-fold from 18 to 42 weeks of age, while no weight change of seminal vesicles was clearly observed in WT controls (Fig. 3B). H&E staining was performed for paraffin-embedded sections of seminal vesicles to determine morphological features and tissue organization. In consistent with the changes in volume and weight of seminal vesicles in TRAMP mice aged from 18 to 42 weeks old, the number of positively stained cells was distinctly increased in histological image of seminal vesicles in 24-week old TRAMP mice and was highly maintained during the growth period of TRAMP mice, whereas the positively stained cells in seminal vesicles of WT controls were kept at a low density during the trial period (Fig. 3C). Normal seminal vesicles are saccular glands composed of central cavity and peripheral glandular including one single layer of columnar epithelium and the stroma layer with smooth muscle cells and collagen fibers 6, 13. In contrast, seminar vesicles of TRAMP mice exhibited a clear papillary-phyllode pattern in structure with multiple alveolar-like spaces from 24 weeks, and were gradually filled by cell proliferation in stroma for the most part with age (Fig. 3C), which demonstrated tumor occurrence and development in seminal vesicles of TRAMP mice from a morphological viewpoint and C57BL/6 TRAMP mice might be a good model in studying seminal vesicle tumors.

**Figure 4:**
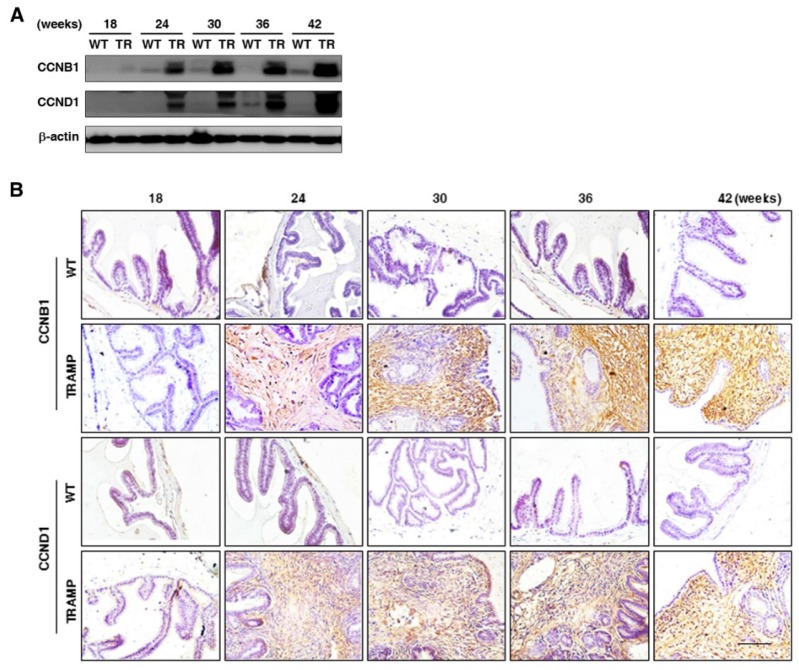
** Comparison for CCNB1 and CCND1 expressions in seminal vesicles between TRAMP and WT mice. (A)** CCNB1 and CCND1 protein levels in seminal vesicles of mice at 12, 24, 36 and 48 weeks of age were measured by Western blotting, seperately, and β-actin level was used as loading control. **(B)** CCNB1 and CCND1 levels were analyzed in paraffin sections of seminal vesicles of TRAMP and WT mice at indicated ages by IHC (Scale bar: 50 μm), respectively. Only representative data from at least 3 independent experiments are shown.

To further explore the potential molecular mechanism of seminal vesicle tumor growth in TRAMP mice, CCNB1 and CCND1 were measured by Western blotting and IHC, respectively. Protein levels of CCNB1 and CCND1 in seminal vesicles of TRAMP mice were found to be dramatically elevated at 24 weeks and continuously increased till the end of trial period, while they were hardly detectable in seminal vesicles of WT groups (Fig. 4A). IHC images also showed that the expressions of CCNB1 and CCND1 were distinctly augmented in seminal vesicle tissues of TRAMP mice starting from 24 weeks compared to WT controls, especially in highly proliferated stroma areas (Fig. 4B). The results marked the tumor formation and progression in seminal vesicles of TRAMP at molecular level since dysregulation of cell cycle was a hallmark of the transformation of normal cells into tumor cells 14.

### P21 and P27 promote cell proliferation and tumorigenesis via accelerating cell cycle progression in seminal vesicles of TRAMP mice

**Figure 5:**
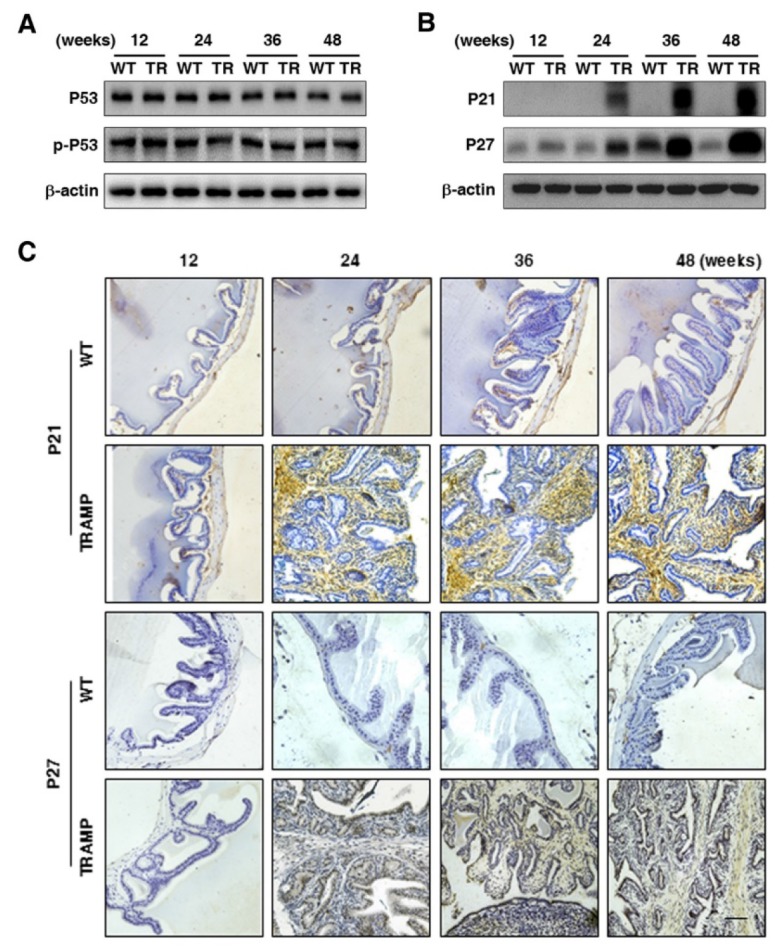
** Comparison for P53, p-P53, P21 and P27 expressions in seminal vesicles between TRAMP and WT mice. (A)** P53 and p-P53 were measured at protein level in seminal vesicles of TRAMP and WT mice at 12, 24, 36 and 48 weeks of age by Western blotting using β-actin level as internal control, which was performed to detect **(B)** P21 and P27 levels as well. **(C)** P21 and P27 were analyzed in paraffin sections of seminal vesicles of TRAMP and WT mice at indicated ages by IHC (Scale bar: 50 μm). Only representative data from at least 3 independent experiments are shown.

To verify the regulatory genes of CCNB1 and CCND1 in seminal vesicle tumors of TRAMP mice, the expressions of cell cycle modulators P53 and p-P53 were analyzed by Western blotting, which displayed that P53 protein level was almost not changed in seminal vesicle tumors of TRAMP mice compared with WT controls, so did p-P53 (Fig. 5A). *P53* is the most commonly silenced or the mutated gene in cancer, where it not only loses its anti-tumor transcriptional activity, but also often acquires oncogenic functions to promote cell proliferation and tumor progression, particularly in *P53* mutational status 15, 16. However, *P53* mutant was not found in seminal vesicle tumors (data not provided). In contrast, P21 and P27 were increased considerably at protein level in seminal vesicles of TRAMP mice starting from 24 weeks while P21 was rarely detectable and P27 was kept at a rather low level in WT controls (Fig. 5B). Similarly, IHC analysis also confirmed that P21 and P27 were substantially increased in seminal vesicles of TRAMP mice compared with WT controls (Fig. 5C).

**Figure 6:**
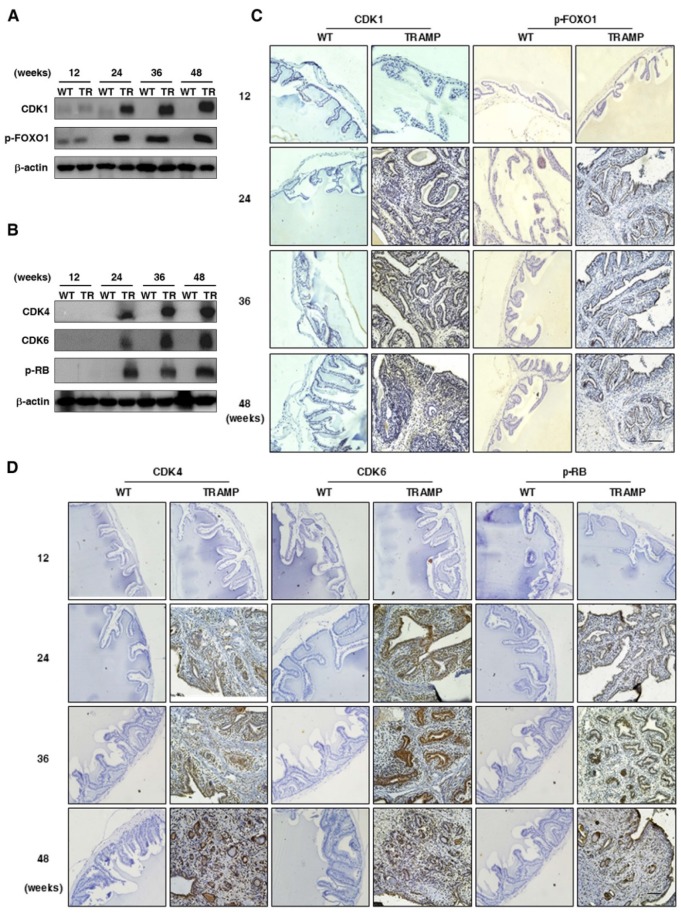
** Comparison for the expressions of P21 and P27 downstream signalings in seminal vesicles between TRAMP and WT mice. (A)** CDK1, p-FOXO1, **(B)** CDK4, CDK6 and p-RB were measured at protein level in seminal vesicles of TRAMP and WT mice at 12, 24, 36 and 48 weeks of age by Western blotting using β-actin level as internal control, separately. **(C)** CDK1, p-FOXO1, **(D)** CDK4, CDK6 and p-RB were analyzed by IHC in paraffin sections of seminal vesicles of TRAMP and WT mice at indicated ages, respectively (Scale bar: 50 μm). Only representative data from at least 3 independent experiments are shown.

It is well known that CCNB1 triggers mitosis in the G2 phase to promote cell proliferation and tumorigenesis by phosphorylation and inhibition of FOXO1 in cancers after binding to CDK1 and initiating the kinase activity 17-19. CCND1 is essential for cell entry into S phase from G1 when associated with CDK4/6, which contributes to the hyperphosphorylation of RB as a tumor suppressor and causes inactivation of its growth-inhibitory function 14, 20, 21. To further verify whether up-regulation of P21 and p27 does not inhibit cell cycle to affect cell proliferation and tumor progression in seminal vesicles of TRAMP mice, CDK1 and downstream FOXO1 phosphorylation were detected by Western blotting. Both of them were found to be largely increased in seminal vesicles of TRAMP mice from 24 weeks to the end of trial period while there were no obvious expressions of them in normal controls (Fig. 6A), so were CDK4, CDK6 and p-RB (Fig. 6B). Subsequent IHC images confirmed similar trends not only for CCND1 and p-FOXO1 expressions (Fig. 6C) but also for CDK4, CDK6 and p-RB expressions in seminal vesicles of TRAMP mice (Fig. 6D). The results demonstrated that P21 and P27 did not play an anti-tumor function in seminar vesicles of TRAMP mice, and they conversely promoted tumor growth.

### EGFR, AKT, STAT3 and EZH2 participate in promoting tumor growth in seminal vesicles of TRAMP mice

**Figure 7:**
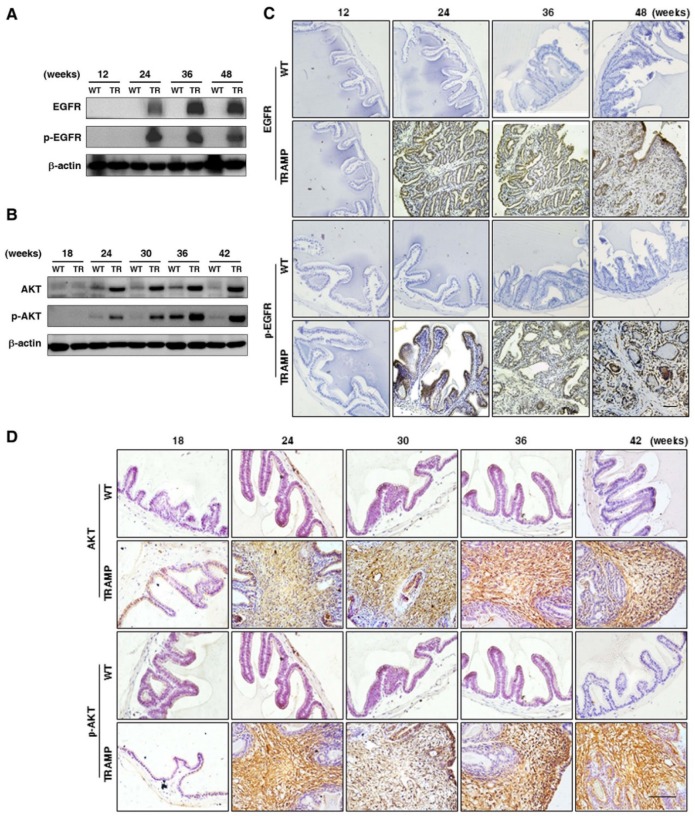
** Comparison for EGFR, AKT and their phosphorylated expressions in seminal vesicles between TRAMP and WT mice. (A)** EGFR, p-EGFR, **(B)** AKT and p-AKT were measured at protein level in seminal vesicles of TRAMP and WT mice at 12, 24, 36 and 48 weeks of age by Western blotting using β-actin level as internal control. **(C)** EGFR, p-EGFR, **(D)** AKT and p-AKT were analyzed by IHC in paraffin sections of seminal vesicles of TRAMP and WT mice at indicated ages, respectively (Scale bar: 50 μm). Only representative data from at least 3 independent experiments are shown.

**Figure 8:**
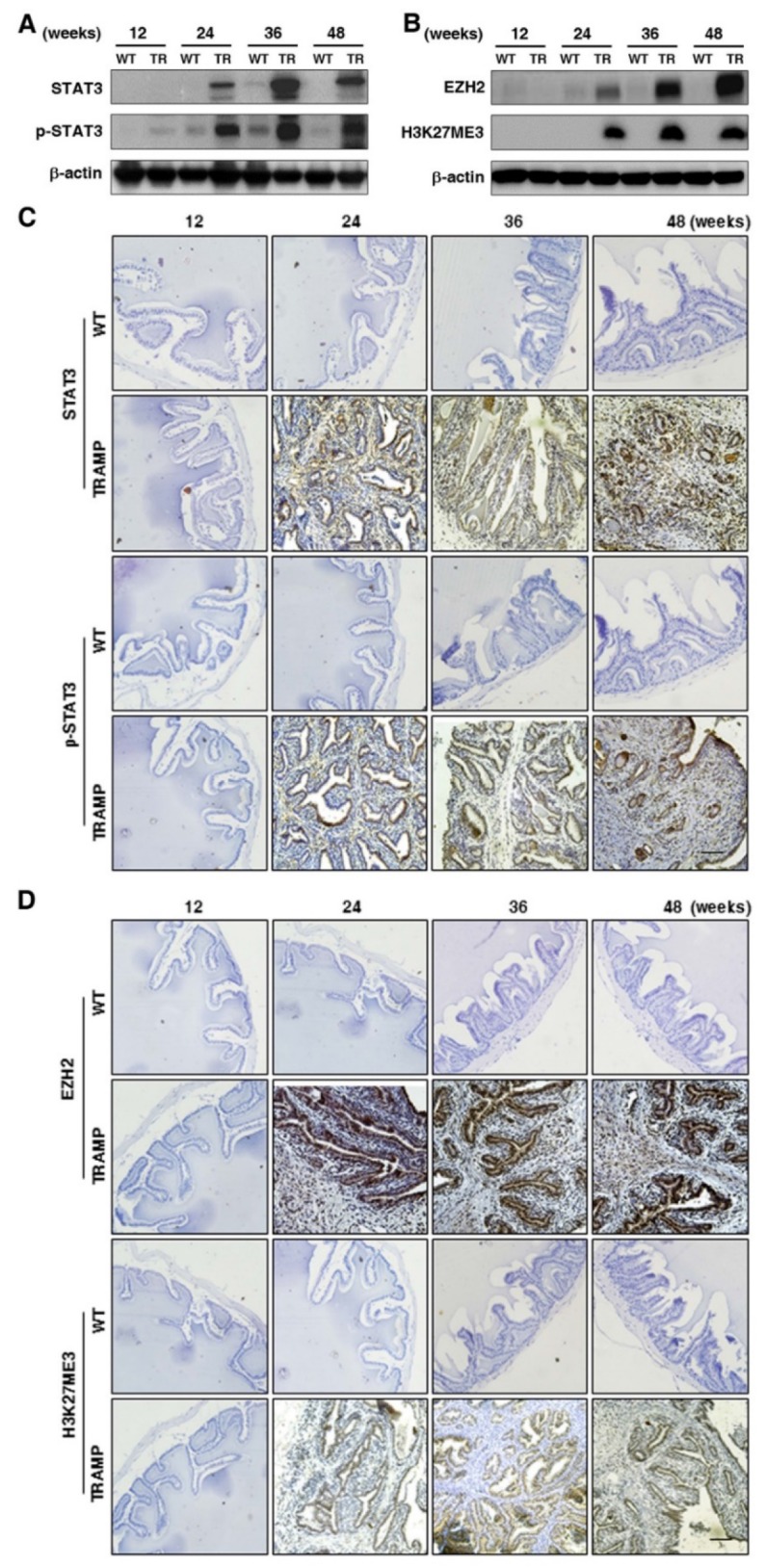
** Comparison for STAT3, p-STAT3, EZH2 and EZH2 mediated H3K27me3 expressions in seminal vesicles between TRAMP and WT mice. (A)** STAT3, p-STAT3, **(B)** EZH2 and H3K27me3 were measured at protein level in seminal vesicles of TRAMP and WT mice at 12, 24, 36 and 48 weeks of age by Western blotting using β-actin level as internal control. **(C)** STAT3, p-STAT3, **(D)** EZH2 and H3K27me3 were analyzed by IHC in paraffin sections of seminal vesicles of TRAMP and WT mice at indicated ages, respectively (Scale bar: 50 μm). Only representative data from at least 3 independent experiments are shown.

To explore the regulation in oncogenic transformation of P21 and P27, EGFR, AKT, STAT3 and EZH2 were measured at protein level by Western blotting. The results displayed that EGFR and p-EGFR (Fig. 7A) were clearly augmented in seminal vesicles of TRAMP mice from 24 weeks and then continuously maintained at a higher level till the end of trial period in comparison with non-transgenic controls, so were AKT and p-AKT (Fig. 7B), which were also confirmed by IHC analyses in seminal vesicle tumors of TRAMP mice (Fig. 7C, D). Moreover, not only STAT3 and p-STAT3 (Fig. 8A) but also EZH2 and EZH2 mediated H3K27me3 (Fig. 8B) had similar trends to be overexpressed in seminal vesicles of TRAMP mice, which were also confirmed by IHC (Fig. 8C, D). The results indicated that EGFR, AKT, STAT3, and EZH2 all play a crucial role in promoting cell proliferation and tumor growth in seminal vesicles of TRAMP mice.

## Discussion

Tumor growth is tightly related to aberrant cellular proliferation, in which various cyclin gene products forming the regulatory subunits of the CDK complexes promote cycle progression from G1, S, G2 to M phases 22, 23. Mammalian cells have at least five cyclin classes (cyclins A to E) reaching maximum abundance at different points in the cell cycle 24. Among them, both of CCNB1 and CCND1 are overexpressed in many cancers 25. The expression and activity of CCND1 and CCNB1 can be blocked by P53 inducing cell cycle inhibitors P21 and/or P27 through transcriptional regulation 26. In this study, CCNB1 and CCND1 were found to be dramatically increased in seminal vesicles of TRAMP mice starting from 24 weeks of age and continuously risen till the end of trial, P21 and P27 expressions had similar increase trends like CCNB1 and CCND1 in seminal vesicles of TRAMP mice, respectively, whereas p53 and p-p53 were constantly expressed during the trial period and almost had no difference with normal controls, which indicated upregulation of p21 and P27 did not result from P53-dependent transcription regulatory function of tumor suppression, i.e., P53 lost tumor suppressor function. Conversely, P21 and P27 might promote seminal vesicle tumor growth, which was confirmed by the increased levels of downstream genes of P21 and P27 signaling such as CDK1-p-FOXO1 and CDK4/CDK6-p-RB.

**Figure 9:**
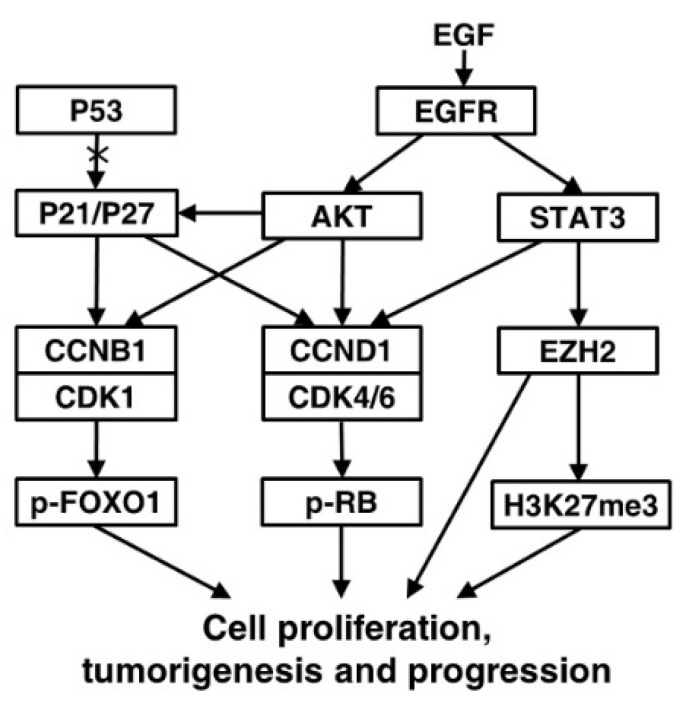
** A proposed signaling network of regulating the formation and development of seminal vesicle tumors in TRAMP mice.** EGFR-AKT pathway mediated P21 and P27 play an important oncogenic role of accelerating cell cycle to promote tumorigenesis and tumor progression in seminal vesicles of TRAMP mice through up-regulation of CCNB1 and CCND1 when P53 function is lost, and other downstream molecules of EGFR such as STAT3 and EZH2 are also associated with promoting cell proliferation in seminal vesicle tumors of TRAMP mice.

Epidermal growth factor receptor (EGFR), a family of tyrosine kinase receptors, is frequently upregulated in human cancers. Upon ligand binding, EGFR may be homodimerized or heterodimerized for autophosphorylation of tyrosine residues to transmit growth factor effect through its diverse downstream signals such as AKT and STAT3 27. AKT phosphorylation can up-regulate the expression of CCNB1 and CCND1 by increasing the transcriptional activity of androgen receptor and inactivating glycogen synthase kinase-3 beta, respectively 28, 29. Importantly, Akt-induced phosphorylation of P21 can enhance P21 stabilization resulting in cytoplasmic localization of P21, and then P21 is transformated into oncogenic protein from nuclear tumor suppressor 30, 31. When WT P53 is absent or damaged, P21 can be functionally influenced by its subcellular localization to act as an oncogenic factor in many cancers 32. During carcinogenesis, such transformation might happened to P27 alike 31. We found AKT and its upstream EGFR were increased in seminal vesicle tumors of TRAMP mice from 24 weeks of age, so were their phosphorylated levels, which might result in oncogenic transformation of P21 and P27 to promote cell proliferation and tumor growth via cell cycle acceleration in WT P53-null seminal vesicles of TRAMP mice. As another downstream gene of EGFR, STAT3 phosphorylation can bind CCND1 promoter to affect the expression and activation of CCND1 for altering cell cycle progression, which can also enhance transcriptional activity of EZH2, a core subunit of polycomb repressive complex 2 (PRC2) 33, 34. EZH2 can catalyze H3K27me3 dependent on PRC2 for transcriptional silencing, methylate non-histone protein substrates for transcriptional silencing or activation, and act as a PRC2-independent co-activator in transcriptional activation, which has been well documented to overexpress in numerous cancers to regulate cell proliferation 35-39. Notably, EZH2 can affect the expressions of cell cycle inhibitors, e.g., *EHZ2* silencing with RNA interference increased P53 and P21 levels to control CCNB1 expression for induction of cell cycle arrest in lung cancer cells, and *EZH2* knock down brought about the increase of P21 and P27 expressions, resulting in CCND1 decrease in non-small cell lung cancer 39, 40. We found both of EZH2 and H2K27me3 were evidently elevated like P21 and P27 in seminal vesicles of TRAMP mice from 24 weeks of age, while STAT3 and its phosphorylated form p-STAT3 was overexpressed, which suggested that STAT3-EZH2 might confer a significant improvement in seminal vesicle tumor cell proliferation of TRAMP mice, and further demonstrated anti-tumor activities of P53, P21 and P27 were lost in seminal vesicles of TRAMP mice. Taken together, our data were recapitulated to unravel molecular mechanism implicated in tumorigenesis and tumor progression in seminal vesicles of TRAMP mice (Fig. 9).

In addition, we found only less than 10% of C57BL/6 TRAMP mice developed prostate tumors while seminal vesicle tumors grew in all of TRAMP mice, suggesting that seminal vesicle tumors was unlikely derived from prostate tumor in TRAMP mice. Meanwhile, more than 60% of macroscopic metastasis occurred in lung and livers of TRAMP mice undergoing sudden death, confirming TRAMP mice death was tightly associated with metastasis of seminal vesicle tumors and reflecting a somewhat greater extent of tumor malignancy in seminal vesicles. CCNB1 and CCND1 expressions were not enough to be a biomarker for malignant seminal vesicle tumors in TRAMP mice though they were statistically distinguishable between malignancy and benign lesion in a series of tumors. P53 lost anti-tumor activity in seminal vesicle tumors of TRAMP mice while P53 expression was retained and not mutated, which was also like in a lot of malignant tumors such as cervical cancer, acute lymphoblastic leukaemia, acute myeloblastic leukaemia, etc 41. Most important of all, it was oncoprotein transformation of P21 and P27 to promote cell cycle acceleration in seminal vesicle tumors leading to uncontrolled cell proliferation and tumor growth under the conditions of lacking WT P53 function. It was reported that Tag interference was necessary to initiate WT P53 function inactivity, but continual Tag level was not required 1, 42. Although Tag was not found to be expressed in a lot of tissues including seminal vesicles but dorsal and ventral prostate tissues of TRAMP mice only when TRAMP model was at first constructed in 1990s, Tag was detected in the stromal cell nuclei of seminal vesicular tumors in 28 week-old TRAMP mice as well as neuroendocrine tumors of the urethra and renal tubulocacinar carcinomas in TRAMP mice afterwards 5. This indicated that oncogenic Tag was not to be completely restricted to express in prostates of TRAMP mice, suggesting P53 lacking WT function in seminal vesicle lesions of TRAMP mice might be caused by Tag oncoprotein expressed in seminal vesicle of TRAMP mice sometime, regardless of the status of Tag expression at any experimental time point selected in the study. Thus, the finding increased new evidence for origin and malignancy of seminal vesicle tumors from a molecular point of view.

In summary, we reported CCNB1 and CCND1 were overexpressed to accelerate cell cycle in seminal vesicle tumors of TRAMP mice from 24 weeks old or so, which might be regulated by EGFR-AKT signaling pathways through oncoprotein transformation of P21 and P27 when P53 has no anti-tumor activity. Meanwhile, STAT3 and EZH2 also promoted tumor growth. This finding unraveled the molecular mechanism associated with carcinogenesis and development in seminal vesicles of TRAMP mice.

## Abbreviations

TRAMP: Transgenic adenocarcinoma mouse prostate
WT: Wild-type
CCN: cyclin
IHC: Immunohistochemistry
CDK: Cyclin-dependent kinase
FOXO1: Forkhead box protein O1
RB: Retinoblastoma-associated protein
EGFR: Epidermal growth factor receptor
AKT: AKT serine/threonine kinase
STAT3: Signal transducer and activator of transcription 3
STAT3: Signal transducer and activator of transcription 3
EZH2: Enhancer of zeste homolog 2
PRC2: Polycomb repressive complex 2
H3K27me3: Trimethylation of histone H3 lysine 27
